# The potential impact of GLS and PDHA1 on tumor immunity and immunotherapy response in LUSC

**DOI:** 10.3389/fgene.2025.1606111

**Published:** 2025-09-19

**Authors:** Tianhe Ling, Jiahui Wu, Ling Xiaohao

**Affiliations:** ^1^ The Tenth Clinical Medical College, Guangzhou University of Chinese Medicine, Zhongshan, China; ^2^ Department of Respiratory and Critical Care Medicine, Zhongshan Hospital of Traditional Chinese Medicine, Zhongshan, China; ^3^ Department of Rehabilitation, Boai Hospital of Zhongshan, Zhongshan, Guangdong, China

**Keywords:** lung squamous cell carcinoma (LUSC), cuproptosis, GLS, PDHA1, immune microenvironment, immunotherapy, metabolic-immune crosstalk

## Abstract

**Background:**

Lung squamous cell carcinoma (LUSC), a therapeutically challenging non-small cell lung cancer (NSCLC) subtype with a poor prognosis, exhibits heterogeneous responses to immunotherapy. Cuproptosis, a recently discovered regulated cell death pathway, has been hypothesised to modulate the tumour immune microenvironment (TIME). Despite the well-established role of PDHA1 as a metabolic regulator, the specific mechanisms by which it interacts with GLS in cuproptosis-mediated immune-metabolic crosstalk remain to be elucidated in LUSC. The present study investigates the manner in which GLS/PDHA1 expression patterns influence TIME composition and contribute to the stratification of immunotherapy responsiveness.

**Methods:**

It was determined that GLS and PDHA1 were the most significant copper oxidation-related genes, due to their highest absolute correlation with the ESTIMATE immune score. A consensus clustering analysis was conducted on a cohort of 501 TCGA-LUSC patients, with the objective of stratifying patients based on GLS/PDHA1 expression levels. Quantitative analysis of immune infiltration was performed using ESTIMATE, CIBERSORT, and ssGSEA methods. The pathway enrichment analysis was conducted using GSEA and WGCNA. A detailed analysis of 17,050 single-cell RNA sequencing (scRNA-seq) data from two LUSC patients was conducted, which revealed unique gene expression patterns. The validity of these findings was confirmed through the integration of four independent GEO cohorts (GSE181043/37745/43580/115457; n = 278).

**Results:**

Consensus clustering delineated two subtypes:Cluster 1 (low GLS/high PDHA1) and Cluster 2 (high GLS/low PDHA1). Cluster two showed enhanced immune infiltration, characterized by: Elevated immune checkpoint expression and Enriched T-cell activation pathways. Validation across four GEO cohorts confirmed Cluster two conserved immune-hot phenotypewith elevated ESTIMATE stromal scores, reduced tumor purity, and activated immune subsets. scRNA-seq identified malignant epithelial cells as the hub of divergent GLS/PDHA1 expression (high GLS/low PDHA1), orchestrating cuproptosis-immunometabolic crosstalk.

**Conclusion:**

GLS and PDHA1 have been proposed as potential prognostic markers for immunotherapy. Targeting cuproptosis has the potential to convert immunologically cold to hot tumours, thereby advancing precision immunotherapy.

## 1 Introduction

Lung cancer is a prevalent clinical malignancy on a global scale. Based on pathogenesis and histological and morphological features, non-small cell lung cancer (NSCLC) can be further classified into three subtypes: lung adenocarcinoma (LUAD), lung squamous cell carcinoma (LUSC) and large cell carcinoma (Miao et al., 2024). Despite advances in various treatments, the 5-year survival rate for patients with LUSC remains low (Siegel et al., 2022). Furthermore, patients with squamous cell lung cancer are less likely to benefit from targeted therapies than those with lung adenocarcinoma (Imyanitov et al., 2021). Consequently, the majority of patients with advanced NSCLC are treated with single-agent immunotherapy or combination chemotherapy, and the effective response to immunotherapy varies somewhat among NSCLC, regardless of programmed death ligand 1 (PD-L1) expression and tumour mutational burden (TMB) (Santini and Hellmann, 2018). Consequently, the identification of genetic biomarkers capable of predicting immunotherapy suitability for LUSC patients is of paramount importance, with the potential to enhance diagnostic accuracy and improve patient prognosis.

The field of cell death research has gained significant importance in the context of lung cancer treatment, as it facilitates a deeper understanding of the biological mechanisms underlying tumours. Furthermore, it offers potential targets for the development of novel therapeutic strategies (Tong et al., 2022). In 2022, Tsvetkov et al. (Tsvetkov et al., 2022a) discovered that the cuproptosis mechanism involves the binding of copper to the direct target of copper (esterified proteins in the TCA cycle) in the mitochondria, leading to excessive aggregation of esterified proteins, massive protein structural disruption, proteotoxic stress and ultimately non-necrotic cell death. cuproptosis-related genes(CRGs) show prognostic significance across cancers (Xie et al., 2023). Given their potential to shape the tumour microenvironment (TME), and that the mechanism of the role of cuproptosis in the development of LUSC is not yet fully understood, it is hypothesised that understanding the relationship between the TME and CRGs may provide insights into new immunotherapeutic strategies.

Recent studies have demonstrated that pyruvate dehydrogenase E1 subunit α1 (PDHA1) is a pivotal cuproptosis gene that is imperative for the reprogramming of glucose metabolism in tumour cells (Deng et al., 2022). In hepatocellular carcinoma (HCC), PDHA1 expression has been found to be predominantly associated with immune cell infiltration and six immune checkpoint-related genes. This has been identified as an independent prognostic indicator for HCC patients (Zhang et al., 2024; Pan et al., 2024; Zhu et al., 2023). Emerging evidence suggests PDHA1’s clinical relevance in multiple myeloma with potential as a therapeutic target (Wang et al., 2025). Glutaminase (GLS), a pivotal gene in the regulation of glutamine catabolism, has garnered significant attention for its role in regulating tumour metabolism and copper-induced cell death (Cluntun et al., 2017). GLS exhibits a close association with various biological functions and pathways, and is predominantly expressed in the liver (Li et al., 2025).

Therefore, this study aims to comprehensively investigate the roles of PDHA1 and GLS in shaping the immune microenvironment of LUSC and their impact on immunotherapy response. Using transcriptomic data from TCGA/GEO and single-cell sequencing, we will stratify patients based on GLS/PDHA1 expression patterns, analyze immune infiltration characteristics, and validate findings in independent cohorts. We hypothesize that distinct GLS/PDHA1 expression profiles may define immunologically divergent subgroups with differential responses to immunotherapy, potentially serving as novel predictive biomarkers for precision immunotherapy in LUSC.

### 1.1 Data sources and pre-processing

A total of sixteen genes associated with cuproptosis were obtained from scientific journal articles (Tsvetkov et al., 2022b). Transcriptomic data and clinical information were downloaded from the TCGA-LUSC project using R software (version 4.4.0) and the R package TCGAbiolinks. Cases with complete clinical information (e.g., age, sex, T stage, N stage, M stage and prognostic information) were included in the analysis. For 496 primary parenchymal tumour samples and 51 paraneoplastic tissue samples, the HTSeq-FPKM data were transformed by log2(FPKM+1) for further analysis and analysed for differences using HTSeq-Counts. Furthermore, expression profiling data was obtained from the GSE181043, GSE37745, GSE43580 and GSE115457 datasets from the Gene Expression Omnibus (GEO) database (https://www.ncbi.nlm.nih.gov/gds/). The latter contained 109, 66, 73 and 30 lung squamous cell carcinoma tissue samples, respectively. Four GEO datasets were subjected to standard processing, comprising:Dimensional integrity validation; Quantile normalization via limma v3.60.6; Cross-dataset batch correction with ComBat (sva v3.56.0) (Leek et al., 2012). Post-correction PCA confirmed effective batch effect removal (Supplementary Figure S1). The integrated expression matrix (n = 278) was used for validation. Finally, the four expression matrices were combined after correction to validate the immune profile of patients with squamous lung cancer.

Single-cell sequencing data were obtained from the Gene Expression Omnibus (GEO) database (https://www.ncbi.nlm.nih.gov/gds/). The dataset contains two single-cell sequencing samples of untreated lung squamous carcinoma tissues, GSM6047623 and GSM6047625, respectively, which were used to analyse the specific expression of GLS and PDHA1 in different cellular subpopulations. Detailed descriptions of the datasets are provided in Table 1. All analyses in this study were performed using R software (version 4.4.0). The Seurat package (version 5.2.1) was utilised to analyse the single-cell RNA sequencing (scRNA-seq) data (Hao et al., 2021). The process of single-cell quality control involves the elimination of cells of substandard quality. This is achieved by implementing a series of criteria, which are designed to identify cells that do not meet the requisite standards. The initial evaluation yielded the detection of 300 genes, indicating a low RNA content. The subsequent analysis identified 7,500 genes, suggesting the presence of potential doublets. Furthermore, the analysis revealed the presence of more than 25% mitochondrial gene-derived UMIs, indicative of apoptotic or dead cells. Subsequent to this, mitochondrial, ribosomal, and hemoglobin genes were removed from the dataset. The final dataset comprised 17,050 cells and 23,842 genes, which were subjected to further analysis.

**TABLE 1:** Basic information of GEO datasets used in the study

GSE series	Tissue	Organism	Sample size	Platform
GSE181043	LUSC tissues	Homo sapiens	109	GPL11154
GSE37745	LUSC tissues	Homo sapiens	66	GPL570
GSE43580	LUSC tissues	Homo sapiens	73	GPL570
GSE115457	LUSC tissues	Homo sapiens	30	GPL570
GSE200972	LUSC tissues	Homo sapiens	2	GPL24676

The HARMONY method (Korsunsky et al., 2019) was employed to address batch effects across the datasets, and the top 2000 highly variable genes were identified using the FindVariableFeatures function in the Seurat package with default parameters. Principal component analysis (PCA) was then performed on these highly variable genes, and Z-score normalization was applied. Dimensionality reduction was performed using Uniform Manifold Approximation and Projection (UMAP). Cluster identification was performed using the FindClusters function, with a resolution of 0.5. The identification of marker genes for each cluster was performed by the Seurat’s FindMarkers function, with a requirement of fold changes greater than 2. The subsequent annotation and visualisation of these marker genes was conducted utilising CellMarker 2.0 (Hu et al., 2023) and prior literature (Wang et al., 2022).

### 1.2 Immune infiltration analysis

To comprehensively characterize the tumor microenvironment (TME) in lung squamous cell carcinoma (LUSC), we employed a multi-faceted computational framework. The ESTIMATE algorithm quantified stromal and immune components via signature gene expression profiles (Yoshihara et al., 2013), yielding four key metrics: stromal score (indicating stromal cell abundance), immune score (reflecting immune cell infiltration level), ESTIMATE score (a combined stromal/immune index), and tumor purity (representing the malignant cell fraction). CIBERSORT deconvolution, constrained by the principle that all cellular fractions sum to unity, inferred the relative proportions of 22 immune cell types from bulk transcriptomes (Newman et al., 2015). Additionally, ssGSEA, implemented through the GSVA R package (Hänzelmann et al., 2013), assessed the enrichment levels of 28 immune cell signatures derived from published gene sets, offering orthogonal validation of immune infiltration patterns. This integrated multi-method approach enabled a robust quantification of TME heterogeneity across LUSC samples.

### 1.3 Consensus clustering based on GLS and PDHA1 expressions

GLS and PDHA1 expression data were extracted and consensus clustering was performed using the R package ConsensusClusterPlus (Wilkerson and Hayes, 2010). The samples were divided into two categories. Survival significance between clusters was analysed using the ‘survival’ and ‘survminer’ packages.

### 1.4 Gene set enrichment analysis (GSEA)

GSEA was performed using the R package clusterProfiler, with the ‘c5. all.v7.0. entrez.gmt’ annotated gene set downloaded from the MSigDB database to identify significant functional differences between the two classes (Yu et al., 2012). Significant pathway enrichment was identified by a normalized enrichment score (|NES| > 1), a P-value <0.05 and an FDR q-value <0.05.

### 1.5 Differentially expressed genes (DEGs) were identified

Expression profiling data (HTSeq-Counts) were analysed using the R package DESeq2 to identify differentially expressed genes between the two classes (Love et al., 2014). The screening criteria applied were as follows: a log2-fold change greater than one and an adjusted P-value less than 0.05.

### 1.6 Weighted gene co-expression network analysis (WGCNA)

We performed WGCNA analysis of differentially expressed genes using the R package WGCNA (Langfe et al., 2008). In order to ensure that the constructed co-expression network was close to a scale-free distribution, a soft power of five was chosen. The analysis yielded six modules, which were then correlated with taxon, stroma score, immune score, ESTIMATE score, and tumor purity. Subsequently, 17 genes were acquired based on module membership (MM) and gene significance (GS) calculations.

### 1.7 Functional enrichment analysis

Gene Ontology (GO) analysis was performed using the R package clusterProfiler to functionally annotate 17 differentially expressed genes. Subsequently, a protein-protein interaction (PPI) network was constructed using the STRING database (Szklarczyk et al., 2019), which integrates both experimentally validated and computationally predicted interactions from diverse sources, though it should be noted that as an uncurated database, some interactions may lack direct experimental verification. Finally, Spearman correlation analysis between gene expression and immune scores (ESTIMATE and ssGSEA) was conducted using the corrplot R package.

### 1.8 Statistical analysis

All statistical analyses were performed using R (v4.4.0) and SPSS (v25.0). Graphical representations were assembled in Adobe Illustrator (CC 2021). Continuous variables were compared using Wilcoxon rank-sum tests, while correlations were assessed via Spearman’s rank correlation coefficient. Categorical clinical characteristics were analyzed by chi-square tests, with Fisher’s exact test applied for small sample sizes. Associations between molecular subtypes and clinical variables were evaluated using multivariate logistic regression. Survival curves were generated by Kaplan-Meier methodology with log-rank testing. For all multiple hypothesis testing scenarios, p-values were adjusted using the Benjamini–Hochberg false discovery rate (FDR) method. All tests were two-sided, with FDR-adjusted q-values <0.05 considered statistically significant.

## 2 Results

### 2.1 Identification of cuproptosis-immunometabolic regulators and patient stratification

**FIGURE 1:**
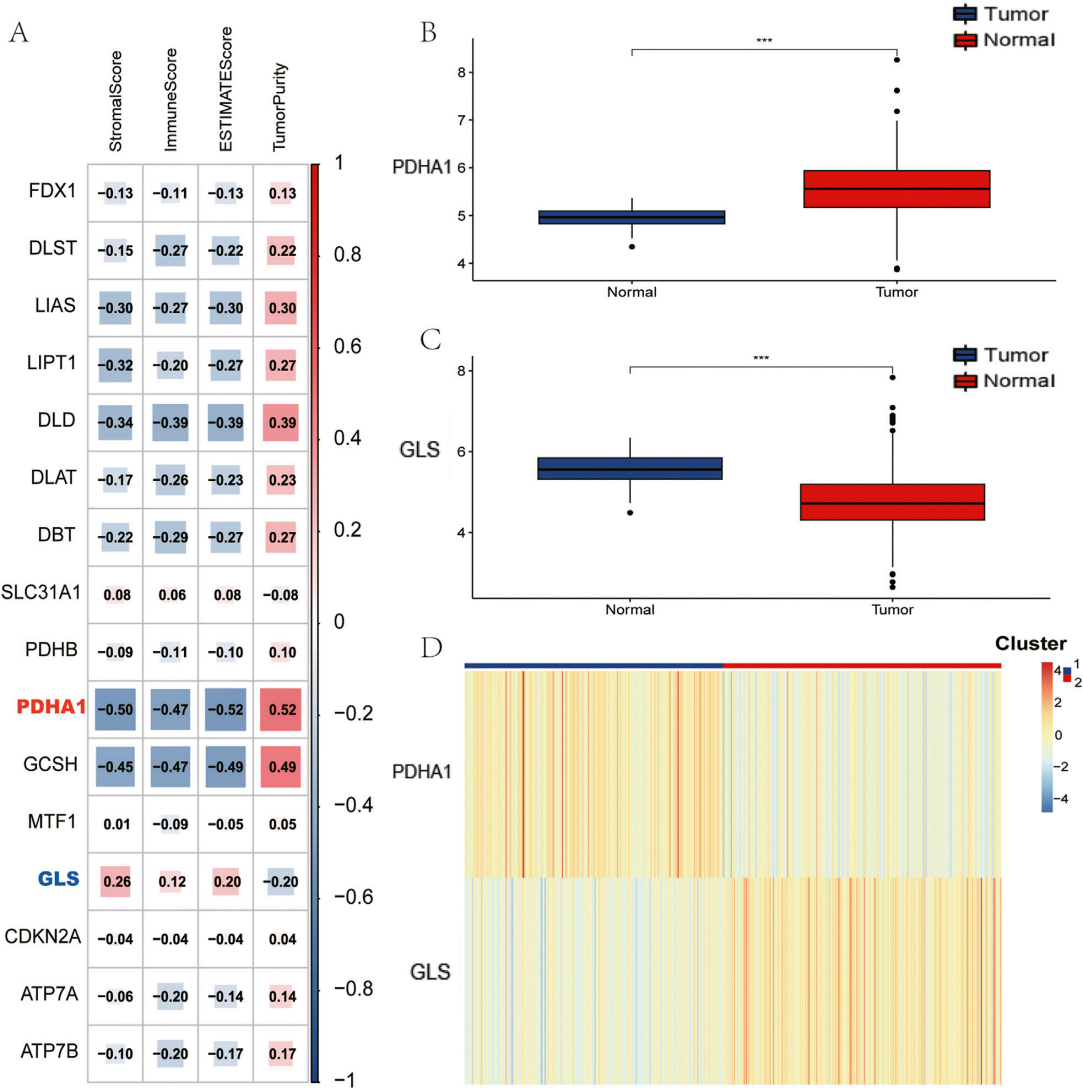
Identification of Cuprptosis-related genes to immune score and clustering of TCGA-LUSC patients based on GLS and PDHA1. **(A)** Association between Cuprptosis-related genes and results of ESTIMATE. **(B)** Comparison of PDHA1 expression between tumor and normal tissues. **(C)** Comparison of GLS expression between tumor and normal tissues. **(D)** TCGA-LUSC patients are divided into two clusters according to GLS and PDHA1.

The analysis was initiated with the calculation of ESTIMATE metrics, with the aim of characterising the composition of the tumour microenvironment across all samples. In order to identify key cuproptosis regulators in LUSC immunity, 16 cuproptosis-associated genes were evaluated, and it was discovered that GLS and PDHA1 exhibited the strongest absolute correlations with immune scores (|ρ| > 0.38, p < 10^−9^; Figure 1A). This prompted the selection of the subjects for further investigation. Subsequent comparison of tumour *versus* normal tissues revealed significant dysregulation: GLS expression was significantly reduced in tumours, while PDHA1 expression was elevated (Figures 1B,C, Wilcoxon p < 0.01). Utilising these findings, a consensus clustering approach was implemented, underpinned by GLS/PDHA1 expression patterns. This approach effectively delineated the 496 TCGA-LUSC patients into two discrete subgroups. As illustrated in Figure 1D, Cluster 1 (n = 239) is distinguished by low GLS/high PDHA1 expression, while Cluster 2 (n = 257) is characterised by high GLS/low PDHA1 expression.

### 2.2 GSEA identification of immune-related pathways

**FIGURE 2:**
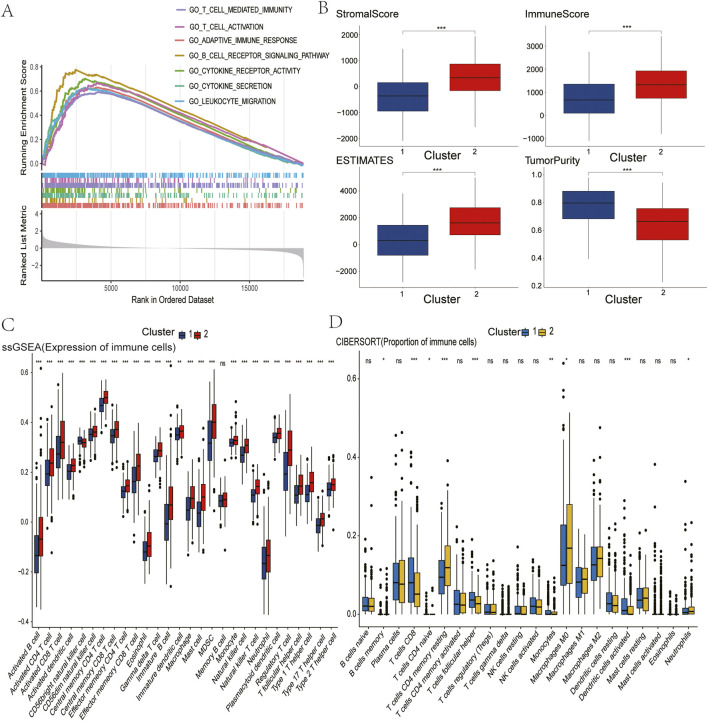
Comparison of immune characteristics between two clusters. **(A)** Comparison of functional enrichment between the two clusters. **(B)** Stromal score, immune score, ESTIMATE score, and tumor purity of the two clusters. **(C)** Proportion of immune cells. **(D)** Expression of immune cells. P values are indicated by asterisks (ns, not significant, *P < 0.05, **P < 0.01, ***P < 0.001).

In order to investigate the functional differences underlying these molecular subgroups, Gene Set Enrichment Analysis (GSEA) was conducted, comparing Cluster 2 with Cluster 1. This analysis revealed significant enrichment (FDR<0.05) of immune-activating pathways in Cluster 2, including adaptive immune response, B/T-cell receptor signaling, cytokine-cytokine receptor interaction, T-cell activation, and leukocyte migration (Figure 2A). These alterations to the pathways suggest that there is enhanced immunogenic potential in tumours with the high GLS/low PDHA1 expression profile.

### 2.3 Comparison of immune infiltration

In pursuance of quantifying immune microenvironment disparities as indicated by pathway analysis, a triad of complementary computational methodologies were employed. The ESTIMATE analysis revealed that Cluster two exhibited significantly elevated stromal, immune, and combined scores, as well as reduced tumour purity, when compared with Cluster 1 (Figure 2B). Furthermore, single-sample GSEA (ssGSEA) revealed the enrichment of 27 immune cell subsets in Cluster 2, including activated B cells, CD4^+^ T cells, CD8^+^ T cells, dendritic cells, and natural killer populations (Figure 2C). These findings were corroborated by CIBERSORT analysis, which revealed increased CD4^+^ T cell and neutrophil infiltration in Cluster 2 (Figure 2D). Collectively, these multilayered assessments establish Cluster 2 as an “immune-hot” phenotype with extensive leukocyte infiltration.

### 2.4 Assessment of immunotherapy sensitivity

**FIGURE 3:**
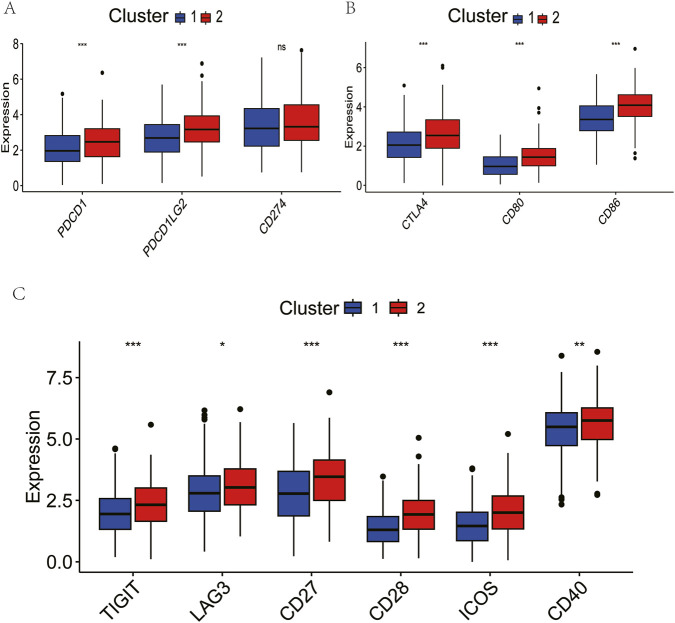
Comparison of clinical immunotherapy drug targets and immunotherapy drug targets in clinical trials for lung squamous cell carcinoma between two clusters. **(A)** PD-1 related. **(B)** CTLA4 related. **(C)** Other Immune Checkpoint. The P values are labeled using asterisks (ns, no significance, **P < 0.01, ***P < 0.001).

The immune-rich phenotype observed in Cluster two was the subject of further investigation in order to ascertain its translational relevance to immunotherapy response. The investigation involved the profiling of key immunomodulatory molecules. A significantly elevated expression of checkpoint inhibitors was detected in Cluster two tumours, including PD-1, PD-L1, CTLA4, CD80/CD86 costimulatory molecules, and emerging targets like TIGIT and LAG3 (Figures 3A–C). Crucially, analysis of clinical trial drug targets revealed that Cluster two exhibited molecular signatures predictive of enhanced response to immune checkpoint blockade therapies, suggesting greater therapeutic vulnerability to these agents compared to Cluster 1.

### 2.5 Identification of cuproptosis-immune hub genes via WGCNA

**FIGURE 4:**
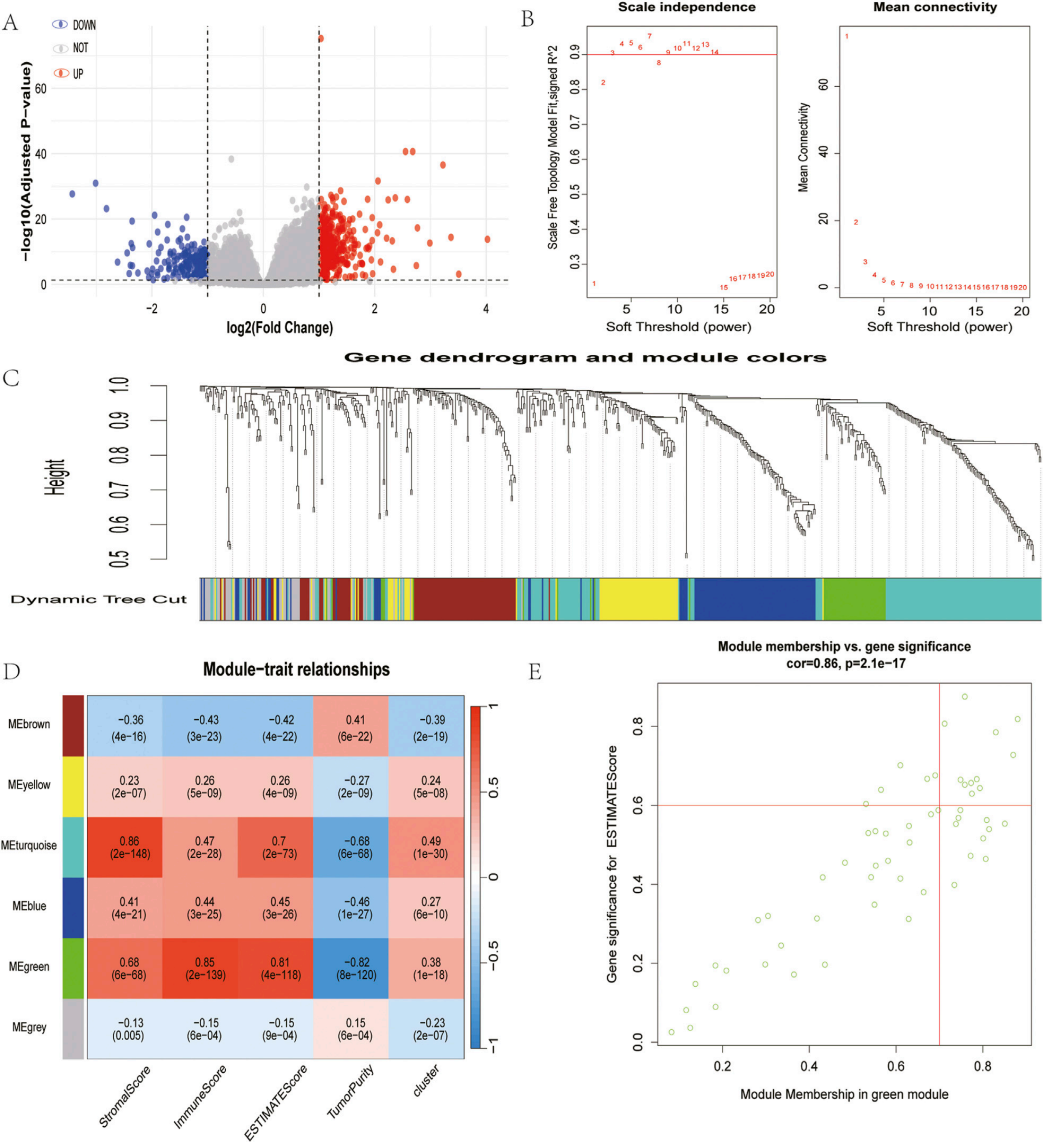
Identification of module genes associated with both clustering and immunity in the WGCNA. **(A)** Volcano plot of differential analysis. **(B)** Analysis of network topology for soft powers. **(C)** Gene dendrogram and module colors. **(D)** Heatmap between module eigengenes and cluster, ESTIMATE results. **(E)** Scatter plot of module eigengenes in the green module.

In an effort to elucidate the molecular network linking copper-induced cell death and immune responses, researchers identified a total of 612 differentially expressed genes across different gene clusters, including 392 genes with upregulation and 220 genes with downregulation. The relevant data are presented in a volcano plot (Figure 4A), and these genes were subsequently used for WGCNA analysis (Figures 4B,C). In view of the correlation between the green module and copper toxicity (R = 0.38, P = 1e-18) and immune traits (R = 0.85, P = 2e-139), the researchers conducted a module-trait correlation analysis (Figure 4D). The results of this analysis indicated that the green module exhibited the strongest dual correlation with both copper toxicity and immune traits. In this pivotal module, 17 high-confidence hub genes were extracted based on stringent connectivity thresholds (MM > 0.7, GS > 0.6). RUBCNL, CSF2RB, SPOCK2, SRGN, EVI2A, TLR10, CCL19, ABI3BP, LTB, CCR4, SELL, FCRL2, CD40LG, TIMD4, TNFRSF13B, FCRLA, and MS4A1) (Figure 4E), thereby identifying core regulatory factors at the interface between copper toxicity and immunity.

### 2.6 Functional characterization of cuproptosis-immune hub genes

**FIGURE 5:**
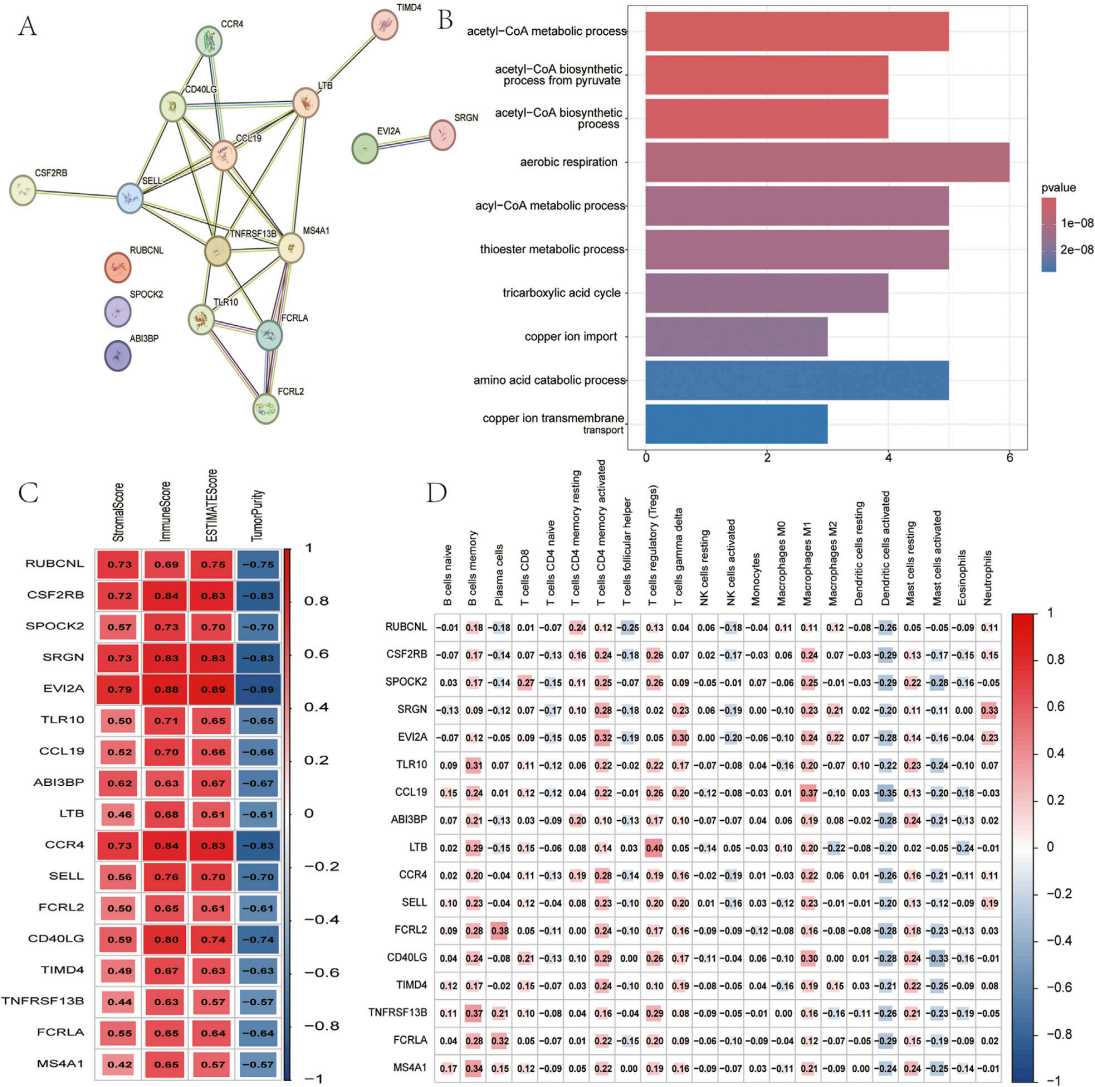
Analysis of 17 hub genes. **(A)** PPI network of hub genes. **(B)** The GO analysis of hub genes. **(C)** Correlation between hub genes and results of ESTIMATE. **(D)** Correlation between hub genes and expression of immune cells (ssGSEA).

To determine their functions, a PPI network was constructed, confirming significant interconnectivity among the 17 hub genes (Figure 5A). Subsequent Gene Ontology (GO) enrichment analysis revealed their strong association with copper ion homeostasis, most notably implicating these genes in acetyl-CoA metabolic processes governing copper ion uptake and transmembrane transport (Figure 5B). Hub genes were found to be significantly associated (p < 0.05) with immune infiltration metrics using both the ESTIMATE and ssGSEA methods (Figures 5C,D). This finding provides strong evidence for a functional link between copper metabolism regulators and tumour immune modulation.

### 2.7 Multi-cohort validation of immune phenotypes

**FIGURE 6:**
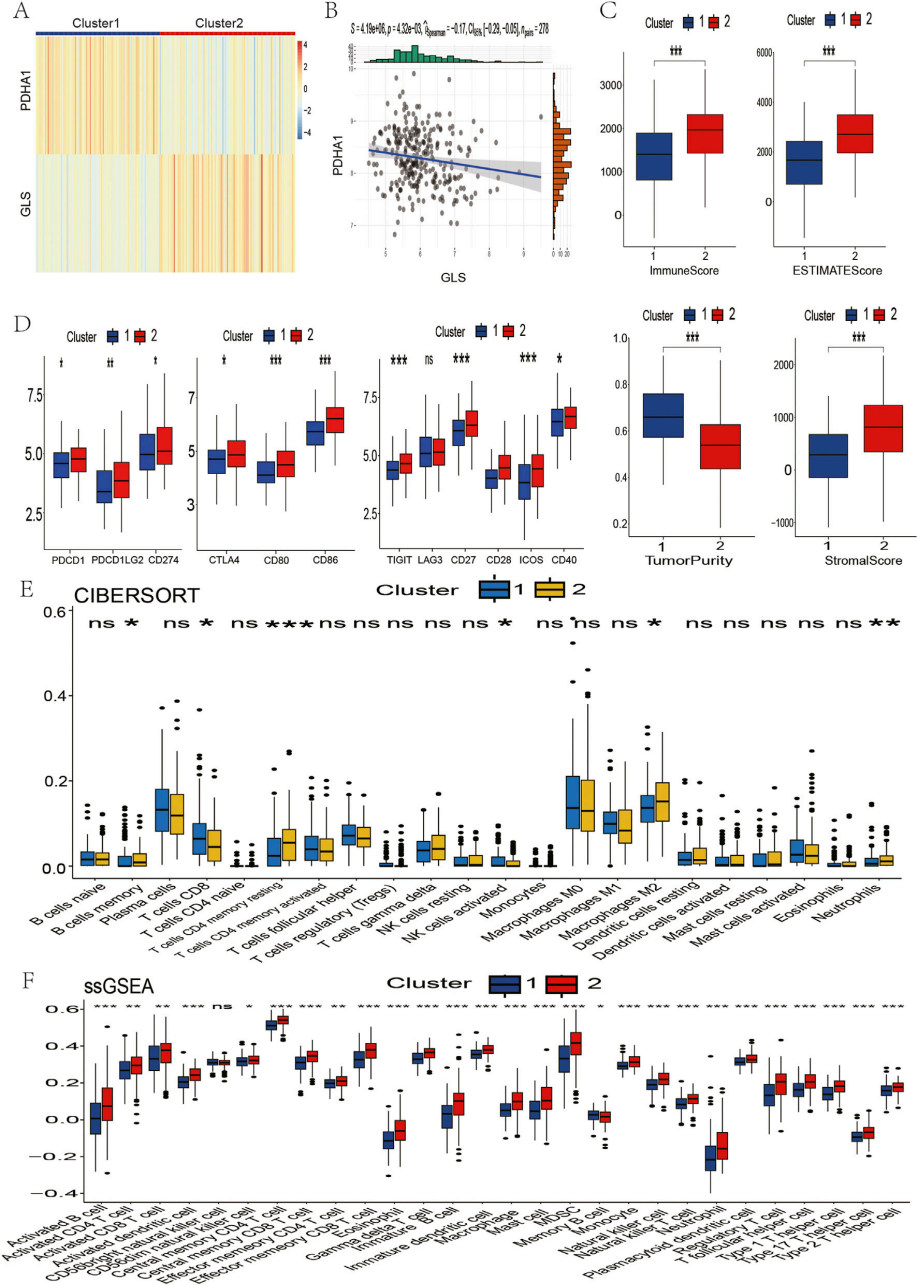
Validation of immune contexture between two clusters in the GSE181043, GSE37745, GSE43580, and GSE115457 datasets. **(A)** Patients in the four datasets were divided into two clusters based on GLS and PDHA1 expression. **(B)** Correlation between GLS and PDHA1 expression in the four GSE datasets. **(C)** Immune score, ESTIMATE score, tumor purity, and stromal score of the two clusters. **(D)** Immune checkpoint inhibitor drug targets. **(E)** Proportion of immune cells. **(F)** Expression of immune cells. P values are indicated by asterisks (ns, not significant, *P < 0.05, **P < 0.01, ***P < 0.001).

LUSC samples were obtained from four GEO datasets, which exhibited batch effects (Supplementary Figure A). Following batch correction, a total of 278 lung squamous cell carcinoma samples were integrated from the four independent GEO datasets. Following the elimination of batch effects, there was a notable enhancement in the consistency of the data distributions across the three datasets. Furthermore, there was a discernible clustering and intertwinement of the data, which is indicative of the efficacy with which batch effects were removed (Supplementary Figure B). In order to validate the reliability of our findings, we employed the same clustering method as TCGA. The resulting subpopulations exhibited expression patterns that were consistent with those observed in TCGA. Specifically, Cluster two demonstrated high GLS and low PDHA1 expression, while Cluster one exhibited the opposite expression pattern (Figure 6A). A notable negative correlation between GLS and PDHA1 expression was observed in the expanded cohort (R = −0.17, p = 0.004; Figure 6B), which was not observed in the discovery cohort. It is imperative to emphasise that all immune parameters (including immune checkpoint expression and infiltration indicators such as ESTIMATE, CIBERSORT, and ssGSEA) exhibited elevated immune activity in Cluster two across all validation datasets (Figures 6C–F). This finding serves to confirm the universality of the proposed stratification framework. In order to validate the robustness of the study results, an independent analysis was conducted of 278 LUSC samples from four GEO datasets using the same clustering method. The resulting subgroups faithfully reproduced the expression patterns observed in TCGA: Cluster two exhibited high GLS/low PDHA1 expression, while Cluster one showed the opposite expression profile (Figure 6A). A notable negative correlation between GLS and PDHA1 expression was observed in the expanded cohort (R = −0.17, p = 0.004; Figure 6B), which was not observed in the discovery cohort. It is imperative to underscore the significance of all immune parameters, including those pertaining to immune checkpoints.

### 2.8 Single-cell resolution of cuproptosis regulator localization

**FIGURE 7:**
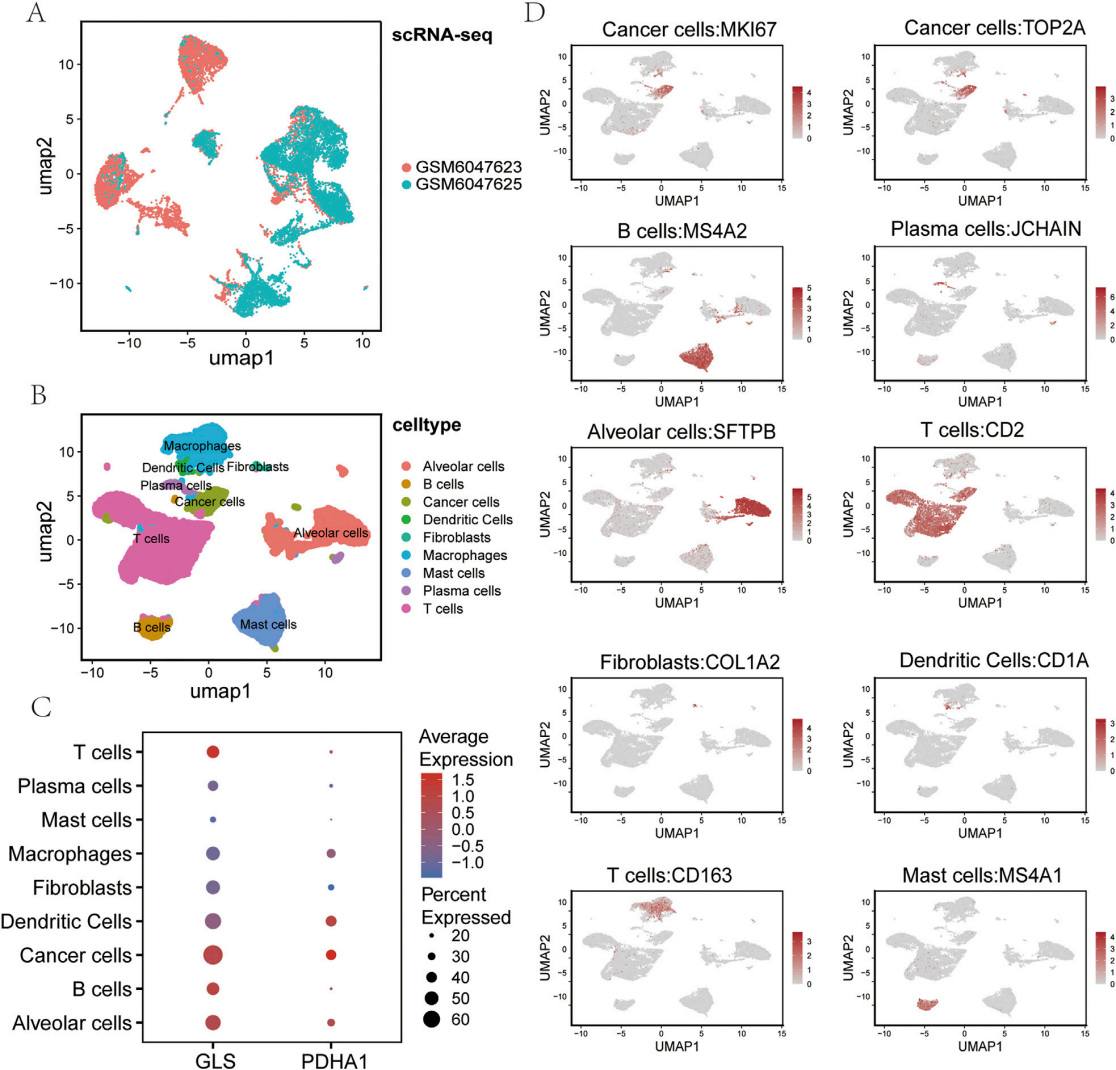
Single-cell RNA sequencing analysis of LUSC. **(A)** UMAP plot showing the sample origin of all single cells from two LUSC tissues. **(B)** UMAP visualization of all cells (n = 23,999) after quality control and dataset integration. Cells are color-coded by major cell types. **(C)** Bubble chart displaying the expression of cuproptosis-related genes GLS and PDHA1 across each major cell type. **(D)** Uniform manifold approximation and projection (UMAP) plot showing the expression of representative markers for each major cell type. LUSC: Lung squamous cell carcinoma UMAP: Uniform manifold approximation and projection.

The tissue utilised for single-cell RNA sequencing (scRNA-seq) was obtained from two patients diagnosed with squamous cell lung cancer. Following comprehensive large-scale screening, 137 million UMIs (unique molecular identifiers) and 23,842 genes were detected in 17,050 cells. Subsequently, the samples from the two lung cancer patients were integrated using uniform distribution approximation and projection mapping (UMAP) (Figure 7A). Batch effects were corrected using a harmonization algorithm. The cells were then divided into nine clusters and classified into known cell lineages, including T cells, B cells, plasma cells, dendritic cells, macrophages, mast cells, fibroblasts, alveolar cells, and cancer cells (Figure 7B). As demonstrated in Figure 7C, the expression levels of select marker genes for each cell type are presented. In order to comprehend the molecular physiological functions of the GLS and PDHA1 genes in the LUSC tumour microenvironment, their localization in LUSC was determined. The results demonstrated that the GLS gene exhibited elevated levels of expression in cancer cells and immune cells (including T cells, B cells, and dendritic cells), while the PDHA1 gene demonstrated comparatively reduced levels of expression in tumor cells (Figure 7D).

## 3 Discussion

LUSC (squamous non-small cell lung cancer) is a histologic subtype of non-small cell lung cancer (NSCLC) that is generally associated with a poorer prognosis and higher mortality rates, largely due to poor therapeutic efficacy and challenging treatment (Lau et al., 2022). Despite the fact that treatments for LUSC include chemotherapy, radiotherapy, and immunotherapy (Forde et al., 2022; Antonia et al., 2018; Felip et al., 2021), the prognosis of patients remains poor (Paz-Ares et al., 2020). A comparison of patients with LUSC and those with lung adenocarcinoma reveals that the former group rarely benefits from targeted therapy, a phenomenon attributable to the low mutation rate and high tumour heterogeneity characteristic of LUSC. Consequently, a combination of chemotherapy and immunotherapy has emerged as a promising therapeutic approach for LUSC management. However, conventional predictive methods, encompassing tumour pathology, tumour staging systems, and PLD-1 expression levels, have proven ineffective in accurately predicting the response to antitumour immunotherapy (Squamous cell lung cancer). Consequently, there is an urgent need to identify new biomarkers and molecular targets to facilitate more precise assessment of the response of LUSC to immunotherapy.

The field of cell death research has been a major area of study in the context of exploring biomarkers. Multiple programmed cell deaths have been identified as valuable indicators in tumour prognosis and immune prediction. cuproptosis, a recently identified copper-dependent mode of cell death, has been shown to be associated with tumourigenesis and progression (Jawed and Bhatti, 2024). Copper ions are one of the trace elements essential for the human body to maintain normal life activities, and an imbalance in intracellular copper ion levels may have an impact on the development and progression of malignant tumours (Nevitt et al., 2012). Tsvetkov et al. (Tsvetkov et al., 2022b) identified 10 CRGs, of which 7 are positively regulated genes, specifically LIAS, FDX1, DLD, LIPT1, PDHB, DLAT, and PDHA1; and three are negatively regulated genes, specifically GLS, MTF1, and CDKN2A.The introduction of the concept of cuproptosis rapidly attracted the attention of medical researchers, and a number of recent studies have found that copper plays an integral role in tumor immunity and antitumor therapy plays an indispensable role (Santini et al., 2014). The concept of cuproptosis, a novel form of programmed cell death contingent on copper ions, has emerged as a promising method for screening active immune responses. This is predicated on the expression of cuproptosis-related genes in conjunction with immune genes, thereby augmenting the infiltration of immune cells within the tumour microenvironment through immune microenvironmental regulation. This regulatory process functions synergistically to enhance the efficacy of anti-tumour immunotherapy (Luo et al., 2023). Li et al. (Li et al., 2023) analysed the transcriptome and single-cell sequencing data of gastric cancer and found that cuproptosis-related genes could be used to classify gastric cancer into two different immune microenvironment subgroups, and established a prognostic model to accurately determine the prognosis of gastric cancer.

Liu et al. (Liu et al., 2022a) from Sun Yat-sen University discovered that differential expression of cuproptosis genes was significantly correlated with the pathological stages of renal clear cell carcinoma and thyroid cancer. Survival analysis demonstrated that the survival risk of patients with renal papillary cell carcinoma was most highly correlated with differential expression of cuproptosis genes. Despite the established correlation between cuproptosis and malignant tumours, the potential of cuproptosis-related gene expression profiles to predict response to immunotherapy in LUSC patients remains to be systematically investigated within the context of squamous lung cancer.

GLS plays a pivotal role in glutamine metabolism and supports tumour growth (Mafra and Dias, 2019; Liu et al., 2022b). Glutaminase (GLS) acts in conjunction with glutamate-derived α-ketoglutarate in the citric acid cycle (TCA) to support cellular energy production and biosynthesis requirements, thereby promoting tumour growth and progression (Liu J. et al., 2022; Saha et al., 2019). Studies have shown that GLS is overexpressed in many malignant tumours (Chen et al., 2022; Gao et al., 2009). In colorectal cancer cells, GLS confers a growth advantage, a pro-tumor microenvironment, and therapeutic resistance by regulating metabolic changes, and colorectal tumors with high expression of glutaminase (GLS) exhibit reduced T-cell infiltration and decreased cytotoxicity, leading to poor clinical prognosis (Yu et al., 2025). Other studies have shown that glutamine metabolism may contribute to the immunosuppressive tumour microenvironment and that inhibition of glutamine metabolism not only suppresses tumour growth but also enhances tumour-specific immunity (Asaka et al., 2024). The present study demonstrated a positive correlation between GLS and immune infiltration of immune cells, and the expression of GLS was significantly elevated in squamous lung cancer. Consequently, it can be hypothesised that elevated GLS expression in tumor cells may be indicative of increased immune cell infiltration within the tumour microenvironment and a more favorable response to antitumor immunotherapy.

Pyruvate dehydrogenase A1 (PDHA1) is a pivotal component of both the tricarboxylic acid cycle and glycolysis, and plays a significant role in cancer-associated metabolic processes. However, its role in LUSC remains to be elucidated. The function of PDHA1 in the maintenance of normal mitochondrial metabolism is well-documented, as is its role in the citric acid cycle (TCA cycle), and its involvement in the regulation, directly or indirectly, of the mitochondrial tricarboxylic acid cycle, which in turn supports cellular energy production (Zhao et al., 2024). The binding of copper ions has been demonstrated to result in the accumulation of lipoylated proteins, which, in turn, has been shown to induce mitochondrial metabolic dysfunction and subsequent cell death (Li). Furthermore, the expression level of PDHA1 has been observed to correlate with the tumor immune microenvironment, and it may influence immune cell infiltration and tumor immune escape mechanisms. Inhibition of PDHA1 has been demonstrated to enhance the malignancy of cancer cells in previous studies (Dupuy et al., 2015; Li et al., 2016). It has been demonstrated that PDHA1 may promote tumour immune escape in certain tumours by regulating metabolism, affecting the efficacy of immunotherapy. RNF4-mediated ubiquitination of PDHA1 has been demonstrated to play a critical role in promoting glycolytic metabolism, proliferation, and metastasis in colorectal cancer, and PDHA1 overexpression has been shown to inhibit CRC cell proliferation, migration, and invasion (Chen et al., 2024).

In this study, we employed consistency clustering analysis to classify LUSC patients in the TCGA database into two categories: cluster 1 (low GLS expression, high PDHA1 expression) and cluster 2 (high GLS expression, low PDHA1 expression). To explore the functional differences between these two subgroups, we performed gene set enrichment analysis (GSEA) using TCGA-LUSC data. The results showed that Cluster two was significantly enriched in immune-related pathways such as adaptive immune response, cell killing, cytokine production, and T-cell activation, suggesting that Cluster 2 has stronger immune response activity. This finding led to the formulation of a hypothesis that Cluster 2 may exhibit more pronounced immune infiltration characteristics. The immune characteristics of the two subpopulations were then assessed by ESTIMATE, CIBERSORT and ssGSEA methods. The stromal score, immune score and composite score of Cluster two were found to be significantly higher than those of Cluster 1, indicating that its tumour immune microenvironment was more active. CIBERSORT analysis revealed that the ratio of helper T cells (CD4^+^) to M0 macrophages was significantly higher in Cluster 2, suggesting that CD4 memory T cells are one of the most important components of the anticancer immune response and are required for the successful elimination of cancer. ssGSEA analysis revealed that Cluster two exhibited a significantly higher proportion of 27 distinct immune cell types, including CD8^+^ T cells, helper T cells (CD4^+^), dendritic cells (DC), natural killer cells (NK), natural killer cells (NKC), natural killer cells (NK), and natural killer cells (NK). The analysis further demonstrated that Cluster two exhibited significantly higher infiltration levels of killer cells (NK), natural killer T cells (NKT), and macrophages. The results obtained demonstrated that cluster 2 (high GLS expression, low PDHA1 expression) exhibited higher levels of immune infiltration and immune checkpoint inhibitor expression in comparison with cluster 1. Consequently, Cluster 2 may exhibit a more robust immune response and be more amenable to immunotherapy.

The tumour microenvironment (TME) is the environment in which a tumour resides and consists of immune cells, stromal cells, extracellular matrix molecules, and various cytokines (Wood et al., 2014). Research has demonstrated the pivotal role of the TME in cancer, with lung cancer exhibiting a high degree of variability in its TME, thereby determining responsiveness and tolerance to immunotherapy (Pitt et al., 2016). The composition of the TME has been demonstrated to be capable of defining the immune phenotype of cancer and may influence the prognosis of cancer patients (Galon and Bruni, 2019). In this study, we sought to explore the potential impact of changes in the expression of cuproptosis-related genes, GLS and PDHA1, on the tumor microenvironment and immune response. These genes, which are closely related to cell metabolism, cuproptosis, and other physiological processes, were selected for analysis through consensus clustering. The findings of this study provide a foundation for further research on the role of the TME in cancer progression and the potential for targeted therapies. Programmed death receptor 1 (PD-1), programmed death ligand 1 (PD-L1), and cytotoxic T-lymphocyte antigen 4 (CTLA4) are FDA-approved immune checkpoint inhibitor (ICI) targets (Karasarides et al., 2022). Lung squamous carcinoma has been observed to evade immune cell attack and develop cancer tolerance by means of immune checkpoint signalling transformation. The application of immune checkpoint inhibitors (ICI) has been demonstrated to reverse immune tolerance and reactivate T cell-mediated cytotoxicity (Kluger et al., 2023). Furthermore, LAG3, TIM3 and TIGIT have been identified as co-inhibitory receptor targets (Yang et al., 2022). In this study, we sought to compare the expression levels of two classes of immunomodulatory targets that have been included in clinical trials for squamous lung cancer. The results demonstrated that the expression levels of the majority of these targets were significantly elevated in Cluster 2.

Subsequently, by means of weighted gene co-expression network analysis (WGCNA), we screened the green modules associated with GLS/PDHA1 and immune score differences, and identified 17 core genes, including CCL19, CD40LG, and LTB. CCL19, a member of the chemokine CC family, is highly expressed in tumours and is able to recruit CD8^+^ T cells and mature dendritic cells (DCs) to infiltrate the tumour core, forming an “immunothermal microenvironment” and promoting the formation of tertiary lymphoid structures (TLS) to enhance the local anti-tumour immune response (Iida and Harada, 2024). CD40LG activates the downstream NF-κB and MAPK signaling pathways by binding to antigen-presenting cells (APCs, e.g., DCs, B-cells, macrophages). MAPK signalling pathways, CD40 agonists have entered clinical trials and significantly enhance the immunotherapeutic effects of lung cancer in combination with PD-1 inhibitors (Tang et al., 2021). LTB, a member of the tumour necrosis factor (TNF) superfamily, promotes the formation of TLS, provides the structural basis for immune surveillance, and synergistically may increase patient sensitivity to immunotherapy (Tertiary lymphoid structures in diseases).

The utilisation of single-cell sequencing technology confers the advantage of enabling the concurrent detection of alterations in cellular composition and gene expression (Wang and Navin, 2015; Qiu et al., 2025). Utilising these findings, we have further elucidated the cell-specific expression patterns of GLS and PDHA1 in the tumour microenvironment of lung squamous carcinoma by employing single-cell sequencing technology. Single-cell transcriptomic data (GSE200972) demonstrated that GLS exhibited significantly elevated expression in cancer cell subpopulations and higher expression levels in immune cells (e.g., T cells, B cells and macrophages) and normal alveolar epithelial cells. Conversely, PDHA1 expression was found to be low across most cell populations, including cancer cells, fibroblasts and immune cells. This differential distribution suggests that the cuproptosis-related gene GLS may drive tumour cell proliferation by regulating glutamine metabolism to provide energy and biosynthetic precursors for tumour proliferation, while high GLS expression in immune cells may enhance their anti-tumour capacity by supporting the metabolic demands of immune cells and maintaining their functional activity (Li et al., 2025; Fang et al., 2023). Conversely, low PDHA1 expression in tumor cells may compel cancer cells to rely on glycolysis, leading to the production of substantial amounts of lactic acid and the formation of an acidic microenvironment. This may, in turn, result in the inhibition of the functional activity of immune cells. Low PDHA1 expression in immune cells may maintain persistent immune cell activity in the tumour microenvironment by reducing mitochondrial oxidative phosphorylation and avoiding immune depletion due to excessive metabolic stress (Mao et al., 2024). By inhibiting mitochondrial oxidative metabolism and reducing the accumulation of immunosuppressive metabolites (e.g., ROS excess) while enhancing glycolysis-dependence, it may indirectly promote immune cell recruitment by remodelling the microenvironment through lactate signalling (Liu et al., 2021). This provides a theoretical basis for predicting immunotherapeutic response in squamous lung cancer using GLS/PDHA1 expression profiling.

In conclusion, Cluster 2 has been shown to behave as a “hot tumour”, indicating the potential for a stronger immune response and a higher probability of response to immunotherapy. Despite the significant advancements in immunotherapy for the treatment of first-line squamous lung cancer, a proportion of patients continue to demonstrate a lack of response to treatment. The combination of immune activation strategies by targeting the cuproptosis pathway and the discovery of new biomarkers predictive of therapeutic response is expected to bring new therapeutic opportunities for LUSC patients.

## 4 Limitations of the present study

The present study revealed the potential role of cuproptosis-related genes (PDHA1 and GLS) in the immune microenvironment of LUSC, but several limitations remain. Firstly, the study relied on the TCGA and GEO databases, which may have limited data representativeness and diversity, and will need to be validated with a wider range of patient samples in the future. Secondly, the methods employed for the assessment of the immune microenvironment (e.g., ESTIMATE, CIBERSORT, and ssGSEA) are based on gene expression, which may not fully reflect the actual status of immune cells and need to be further validated by experimental methods such as flow cytometry. Thirdly, the study was a correlation analysis, which did not clarify the causal relationship between cuproptosis genes and immunotherapeutic response, and this relationship needs to be further explored through animal models and clinical trials. The interaction between the cuproptosis pathway and other metabolic pathways has not been fully explored, and future studies could analyse the effects of their synergistic effects on immune escape. Finally, the validation of clinical samples is lacking, and it is recommended that future studies be further analysed in conjunction with clinical data. In conclusion, while this study has yielded novel insights, further experimental evidence and clinical validation are necessary to more firmly substantiate the potential of cuproptosis-related genes in LUSC immunotherapy.

## 5 Conclusion

The present study, we modelled for the first time the dual roles of cuproptosis-related genes GLS and PDHA1 in the regulation of the immune microenvironment of LUSC, both through metabolic reprogramming that induces immunogenic death, and through a network of core immune genes that shapes the “hot tumour” phenotype. By employing a clustering approach that combined TCGA-LUSC and GSE181043, GSE37745, GSE43580, and GSE115457 patients based on GLS/PDHA1 expression, it was observed that Cluster 2 (high GLS expression, low PDHA1 expression) exhibited increased immune cell infiltration, ICI target expression, and the green module core genes (CCL19, CD40LG, LTB) can assist in evaluating tumour immunoreactivity and therapeutic sensitivity, which provides the theoretical basis and translational direction of precision immunotherapy for LUSC, and is expected to break through the current therapeutic bottlenecks and improve the prognosis of patients.

## Data Availability

The original contributions presented in the study are included in the article/Supplementary Material, further inquiries can be directed to the corresponding author.
