# Supplementary figures and images for: The potential impact of GLS and PDHA1 on tumor immunity and immunotherapy response in LUSC
Source: Front Genet. 2025 Sep 19;16:1606111. doi: 10.3389/fgene.2025.1606111 (PMC12492448; doi:10.3389/fgene.2025.1606111)

Supplementary Figure A

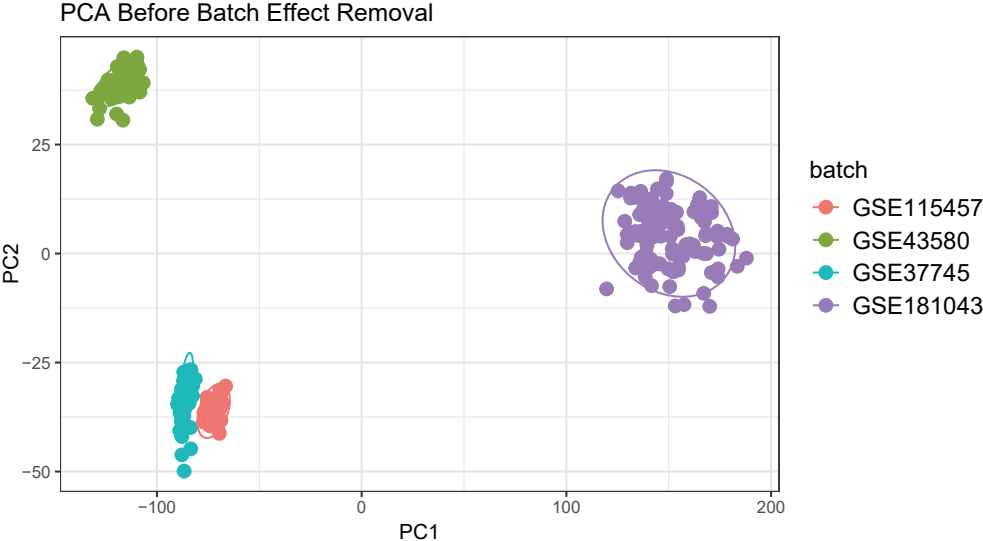

Supplementary Figure B

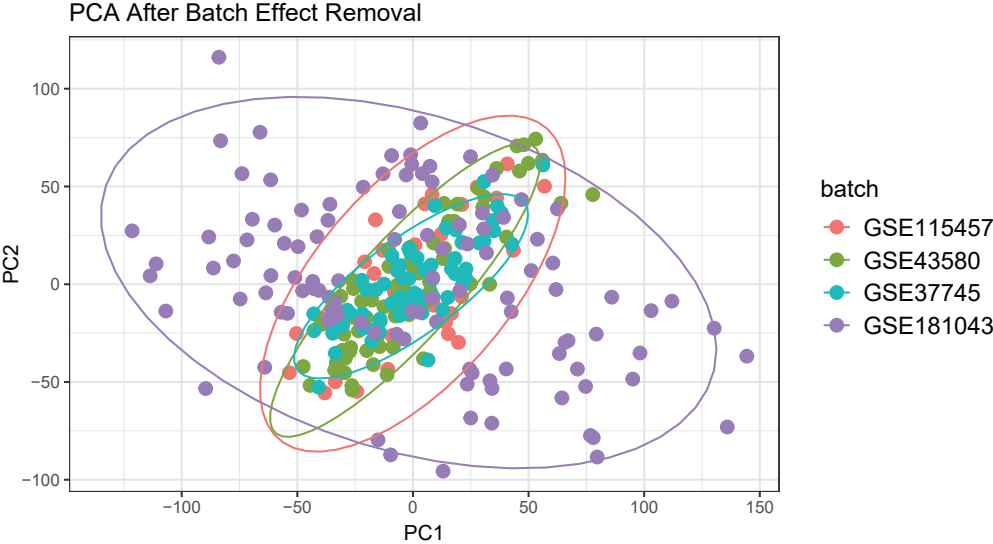

Supplement: Supplementary file 1 [file DataSheet1.pdf]
